# Roles of mannosylerythritol lipid-B components in antimicrobial activity against bovine mastitis-causing *Staphylococcus aureus*

**DOI:** 10.1007/s11274-022-03243-2

**Published:** 2022-02-12

**Authors:** Shinya Yamauchi, Mutsumi Furukawa, Akio Kawahara, Tomohiro Sugahara, Shuhei Yamamoto, Masao Kitabayashi, Atsushi Sogabe, So Shimoda, Eiji Hata, Kouichi Watanabe, Hiroshi Yoneyama, Hisashi Aso, Tomonori Nochi

**Affiliations:** 1 International Education and Research Center for Food and Agricultural Immunology, Graduate School of Agricultural Science, Tohoku University, 468-1 Aoba, Aramaki, Aoba-ku, Sendai, Miyagi 980-8572 Japan; 2 Laboratory of Functional Morphology, Graduate School of Agricultural Science, Tohoku University, Sendai, Miyagi 980-8572 Japan; 3 Toyobo Co., Ltd. Tsuruga Institute of Biotechnology, Fukui, 914-0047 Japan; 4 Toyobo Co., Ltd. Biochemical Department, Osaka, 530-8230 Japan; 5 Division of Bacterial and Parasitic Disease, National Institute of Animal Health, Bacterial Pathogenesis Research Unit, National Agriculture and Food Research Organization, Tsukuba, Ibaraki 305-0856 Japan; 6 Laboratory of Animal Microbiology, Graduate School of Agricultural Science, Tohoku University, Sendai, Miyagi 980-8572 Japan; 7 Laboratory of Animal Health Science, Graduate School of Agricultural Science, Tohoku University, Sendai, Miyagi 980-8572 Japan; 8 Division of Mucosal Vaccines, International Research and Development Center for Mucosal Vaccines, The Institute of Medical Science, The University of Tokyo, Tokyo, 108-8639 Japan

**Keywords:** Mannosylerythritol lipid-B (MEL-B), Bio-surfactant, *S. aureus*, Mastitis, Dairy cattle

## Abstract

Mannosylerythritol lipid-B (MEL-B), which comprises ester-bonded hydrophilic ME and hydrophobic fatty acids, is a bio-surfactant with various unique properties, including antimicrobial activity against most gram-positive bacteria. The gram-positive *Staphylococcus aureus* is a causative pathogen of dairy cattle mastitis, which results in considerable economic loss in the dairy industry. Here, we demonstrate the efficacy of MEL-B as a disinfectant against bovine-derived *S. aureus* and elucidate a mechanism of action of MEL-B in the inhibition of bacterial growth. The growth of bovine mastitis causative *S. aureus* BM1006 was inhibited when cultured with MEL-B above 10 ppm. The activity of MEL-B required fatty acids (i.e., caprylic and myristoleic acids) as ME, the component of MEL-B lacking fatty acids, did not inhibit the growth of *S. aureus* even at high concentrations. Importantly, ME-bound fatty acids effectively inhibited the growth of *S. aureus* when compared with free fatty acids. Specifically, the concentrations of ME-bound fatty acids and free caprylic and myristoleic acids required to inhibit the growth of *S. aureus* were 10, 1442, and 226 ppm, respectively. The involvement of ME in the antimicrobial activity of MEL-B was confirmed by digestion of MEL-B with alkali, which dissociated ME and fatty acids. These results indicated that a mechanism of action of MEL-B in inhibiting the growth of *S. aureus* could be explained by the effective transporting of antimicrobial fatty acids to the bacterial surface via hydrophilic ME.

## Introduction

In the livestock industry, large amounts of antibiotics have been extensively used to cure domestic animals suffering from infectious diseases such as mastitis, diarrhea, and pneumonia (Berendsen et al. 2015; Kromker and Leimbach 2017). Livestock are also administered various antibiotics as feed additives to gain body weight rapidly; however, most countries recently proposed to discontinue using antibiotics as feed additives because of multiple safety concerns (Maron et al. 2013). The most serious issue caused by excess use of antibiotics is the development of antimicrobial-resistant (AMR) bacteria that may then infect human beings (Graham et al. 2009; Lee 2003). The number of human deaths predicted in 2050 due to AMR infection has been estimated to be over 10 million if no actions are taken at the current time (de Kraker et al. 2016). Considering that contagious bacteria can spread globally, the idea of “One Health” that pursues the health of animals, humans, and the environment without the use of excess antibiotics has been widely accepted (Collignon 2015; McEwen and Collignon 2018).

Mastitis is caused primarily by infection with pathogens in the udder of mammals such as dairy cattle (Schrick et al. 2001). Bovine mastitis can be divided into two types based on the classification of the causative bacteria. One type is caused environmentally (so-called environmental mastitis) by infection of bacteria that are commonly found in farms, such as environmental streptococci and *Escherichia coli*. The other type is infectious mastitis, which is mostly caused by *Staphylococcus aureus* (Hospido and Sonesson 2005). Of note, methicillin-resistant *S. aureus* (MRSA), a well-known AMR, has been frequently isolated from milk of dairy cattle with infectious mastitis (Vanderhaeghen et al. 2010). *S. aureus* forms a biofilm to facilitate infection and may survive latently in target cells, such as macrophages, by escaping from the host immune machinery (Scherr et al. 2015; Thurlow et al. 2011). Intracellular *S. aureus* is believed to account for why dairy cattle with subclinical mastitis cause large economical loss due to the reduction of milk production (Almeida et al. 1996; Sinha et al. 2014). Therefore, the development of novel strategies that can prevent infection by *S. aureus* in dairy cattle without using antibiotics not only contributes to “One health,” especially in suppressing the outbreak of AMRs such as MRSA but also facilitates reducing the economic loss caused by both clinical and subclinical mastitis (Sinha et al. 2014).

Mannosylerythritol lipid B (MEL-B) is a glycolipid bio-surfactant produced by the cultivation of *Pseudozyma tsukubaensis* (NBRC1940) in the presence of olive oil (Fukuoka et al. 2008). MEL-B is composed of hydrophilic 4-*O*-β-d-mannopyranosyl-d-erythritol (ME) and two types of hydrophobic fatty acids (e.g., caprylic acid and myristoleic acid in most cases) (Yamamoto et al. 2012). MEL-B possesses several biological characteristics because of the amphiphilic properties and is therefore attracting attention in various fields (Coelho et al. 2020). For instance, MEL-B has been commercialized in the cosmetic industry as a natural ingredient with a moisturizing effect, clearly indicating that there are no safety concerns (Yamamoto et al. 2012). Importantly, the properties of MEL-B include antibacterial activity against most gram-positive bacteria (Kitamoto et al. 1993); however, the mechanism of action is yet to be identified. Furthermore, the inhibitory effect of MEL-B on the growth of *S. aureus* strains derived from dairy cattle has not yet been investigated.

Here, we show that MEL-B inhibits the growth of bovine mastitis causative *S. aureus* BM1006 and demonstrate that the hydrophilic ME acts as a delivery vehicle to transfer the antimicrobial fatty acids that affect bacterial growth. These results indicate that as a bio-surfactant, MEL-B could be useful as a possible disinfectant capable of suppressing outbreaks of *S. aureus* in dairy farms.

## Materials and methods

### MEL-B, ME, and fatty acids

*Pseudozyma tsukubaensis* (NBRC1940) was cultured in production medium that contains 10% (v/v) olive oil, 0.1% (w/v) yeast extract, 4% (w/v) glucose, 0.3% (w/v) NaNO_3_, 0.03% (w/v) MgSO_4_·7 H_2_O, and 0.03% (w/v) KH_2_PO_4_ at 25 °C on a rotary shaker (180 rpm) for 7 days to generate MEL-B, which was comprised ME and fatty acids, mainly caprylic (C8:0) and myristoleic (C14:1) acids. Purification of MEL-B from the culture medium of *P. tsukubaensis* was performed using high-performance liquid chromatography (HPLC) on a silica gel column. A portion of purified MEL-B was reacted with 4-*N*-chloroformylmethyl-*N*-methyl(amino)-7-nitro-2,1,3-benzoxadiazole (NBD), a fluorescent reagent with λ_ex_ = 470 nm and λ_em_ = 540 nm, in dry acetone for 60 min to synthesize NBD-labeled MEL-B. ME was synthesized according to the previous study (Fukuoka et al. 2008).

### Bacterial strains and culture

*S. aureus* BM1006 (MAFF913131), *S. aureus* JE2 (NR-46543; MRSA) and *E. coli* JM109 (ATCC53323) were used in this study. All bacteria were cultured overnight in Trypto-Soya (TS) broth (Nissui) at 37 °C on a rotary shaker (120 rpm). To obtain the numbers of colony-forming units (CFUs), 30 μL of pre-cultured *S. aureus* BM1006 and *E. coli* JM109 containing 5.4 × 10^7^ CFU and 4.8 × 10^7^ CFU, respectively, were added to fresh TS broth (3 mL) containing MEL-B at various concentrations (0, 0.1, 1, 10, 100, and 1000 ppm) to address the effect of MEL-B on bacterial growth for 24 h at 37 °C. Bacterial growth was assessed by measuring the optical density of the culture medium at 660 nm (OD_660_). Precultured *S. aureus* BM1006 or *S. aureus* JE2 (10 μL containing 5.0 × 10^4^ CFU) was added to fresh Mueller–Hinton broth (90 μL) containing MEL-B at various concentrations (0, 0.5, 1, 2, 4, 8, 16, 32, 64, 128, 256, and 512 ppm) in individual wells in 96-well plates. After culturing overnight, the OD_660_ was measured using a 20 × diluted culture medium to assess the effect of MEL-B on bacterial growth. In addition, *S. aureus* was cultured in TS broth containing ME at various concentrations (0.1, 1, 10, 100, and 1000 ppm). *S. aureus* was also cultured for 5 h in the presence of either caprylic acid (144.2 and 1442 ppm) or myristoleic acid (22.6 and 226 ppm). Survival of bacteria in the presence of either MEL-B, ME, caprylic, or myristoleic acid was determined by obtaining the numbers of CFUs. Aliquots of bacterial broth were collected and seeded on TS agar plates (three replicates) after dilution with saline to obtain CFUs.

### Binding analysis

*S. aureus* BM1006 and *E. coli* JM109 were treated with NBD-labeled MEL-B (NBD-MEL-B) at various concentrations (0, 0.1, 1, 10, and 100 ppm) for 30 min at room temperature (RT). After washing three times with phosphate-buffered saline, bacteria were fixed in 4% (w/v) of paraformaldehyde (Nacalai Tesque) for 30 min at RT and affixed on glass slides for fluorescence microscopy (BZ-9000, Keyence). Flow cytometry analysis (Accuri C6, DB) was also used to assess the mean fluorescence intensity of bacteria.

### Scanning electron microscopy analysis

The effect of MEL-B on the surface structure of *S. aureus* BM1006 was investigated by scanning electron microscopy (SEM) analysis. Specifically, bacteria were cultured for 0.5, 2, 5, and 24 h with or without 100 ppm of MEL-B or were treated with 100 U/mL of lysostaphin (WAKO) for 30 min at RT. Bacteria were fixed in 2.5% (v/v) of glutaraldehyde (Nacalai Tesque) for 1 h at 4 °C. After washing, bacteria were affixed to glass slides, coated with platinum and palladium, and analyzed using an SEM SU8000 (Hitachi) at 3.0 kV.

### HPLC analysis

To quantify MEL-B contained in TS broth during the culture of *S. aureus*, HPLC was performed. Culture medium (250 μL) collected after 0.5, 2, 5, and 24 h of culturing was mixed with ethyl acetate (500 μL) for 3 min, and the supernatant was collected by centrifugation (13,000 rpm, 4 °C, 3 min). The process was repeated three times, and MEL-B was extracted by evaporating from the supernatant and dissolved in chloroform. Specifically, the quantification of the extracellular glycolipids was performed using the HPLC of the MEL-B extracts loaded onto a silica gel column (Inertsil SIL 100 A 5 μm, 4.6 × 250 mm; GL Science, Japan) using chloroform/methanol as the solvent system with a gradient flow (1 mL/min) controlled from 100:0 to 0:100. A low-temperature evaporate light-scattering detector (Shimadzu, Kyoto, Japan) was used for detecting MEL-B.

### Analysis of MEL-B components on the antimicrobial activity

MEL-B (2000 ppm) was digested by treatment with 1 M NaOH for 1 h at RT to cleave the ester bonds between ME, caprylic acid, and myristoleic acid; the digested mixture was then neutralized with 1 M of HCl. *S. aureus* BM1006 was then cultured with 10 ppm of alkali-treated or non-treated intact MEL-B. *S. aureus* was also cultured with either 10 ppm MEL-B or an equivalent mixture of 4.3 ppm ME, 2.2 ppm caprylic acid, and 3.4 ppm myristoleic acid to address the role of ester bonds of MEL-B in inhibiting the growth of *S. aureus*. After 5 h, bacterial survival was determined via the CFU count.

### Statistics

Statistical analyses were conducted using one-way analysis of variance (ANOVA) with the Kruskal–Wallis test and two-way ANOVA with Tukey’s multiple comparison test using Prism 7 (GraphPad).

## Results

### MEL-B inhibits the growth of bovine mastitis causative *S. aureus* strain BM1006

**Fig. 1:**
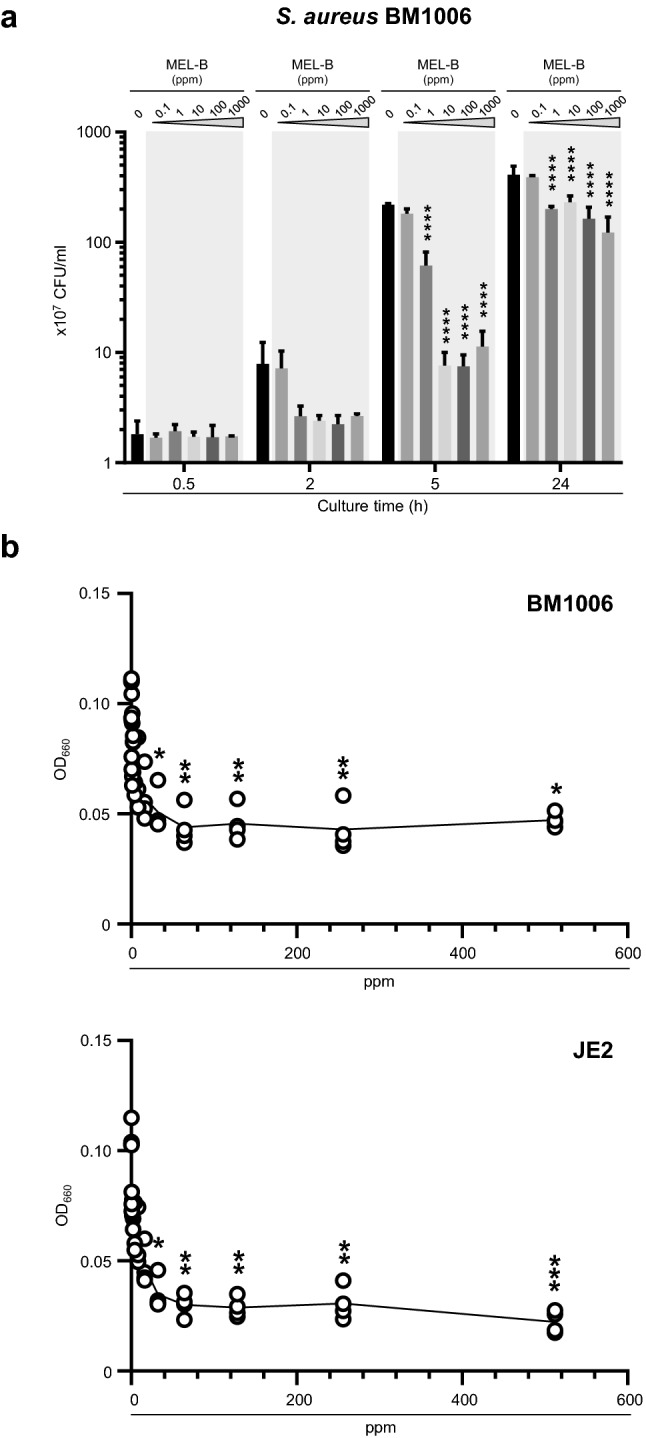
MEL-B inhibits the growth of *Staphylococcus aureus *in vitro. **a** Bovine-derived *S. aureus* BM1006 was cultured in the presence of MEL-B at 0, 0.1, 1, 10, 100, or 1000 ppm for 0.5, 2, 5, and 24 h. The effect of MEL-B on the growth of *S. aureus* was assessed by counting colony-forming units (CFUs). Growth was significantly delayed when cultured with MEL-B above 10 ppm. **b** The efficacy of MEL-B was also confirmed using strains BM1006 and JE2 after culturing overnight by investigating optical density of culture medium measured at 660 nm (OD_660_). The OD_660_ values were significantly lower when both JE2 and BM1006 were cultured in the presence of MEL-B between 32 to 512 ppm in comparison with those in the absence of MEL-B. Experiments were performed in triplicate, and the mean ± standard error of the mean obtained from representative data is shown. Statistical analyses were conducted using two-way ANOVA with Tukey’s multiple comparison test (**a**), and one-way ANOVA with the Kruskal–Wallis test (vs 0 ppm) (**b**). *p < 0.05, **p < 0.01, ***p < 0.001 ****p < 0.0001

**Fig. 2:**
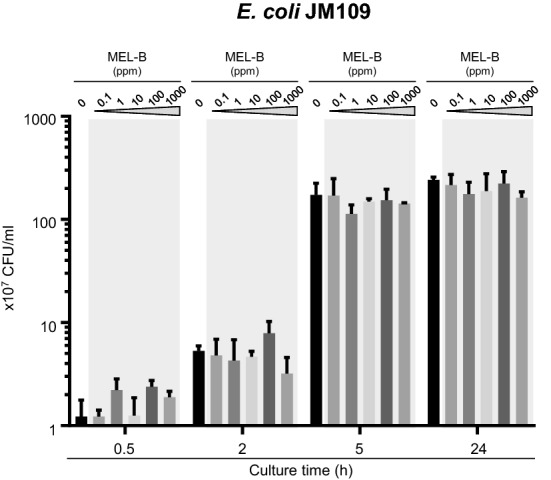
MEL-B does not inhibit the growth of *Escherichia coli *in vitro. *E. coli* JM109, a representative of gram-negative bacterium, was cultured in the presence of MEL-B at 0, 0.1, 1, 10, 100, or 1000 ppm for 0.5, 2, 5, and 24 h. The effect of MEL-B on the growth of *E. coli* was assessed by counting colony-forming units (CFUs). MEL-B did not affect the growth of *E. coli* even at high concentrations. Experiments were performed in triplicate, and the mean ± standard error of the mean obtained from representative data is shown. Statistical analyses were conducted using two-way ANOVA with Tukey’s multiple comparison test

We first sought to demonstrate the efficacy of MEL-B for addressing the roles of MEL-B as a possible disinfectant for use with dairy cattle in their breeding environment. Bovine milk-derived *S. aureus* BM1006, a bovine mastitis causative bacterium (Kiku et al. 2016), was cultured in vitro in the presence of MEL-B at 0, 0.1, 1, 10, 100, and 1000 ppm, and the influence of MEL-B on the growth of *S. aureus* (via CFU counts) was monitored at 0.5, 2, 5, and 24 h. The growth of *S. aureus* was significantly delayed when cultured with MEL-B, and the minimum concentration of MEL-B required for sufficient inhibition of bacterial growth was 10 ppm (Fig. 1a). The CFU counts of *S. aureus* cultured for 5 h in the presence or absence of 10 ppm MEL-B were 7.6 ± 2.4 × 10^7^ and 2.2 ± 0.4 × 10^9^, respectively, indicating that the number of viable *S. aureus* bacteria was reduced by more than 28-fold following exposure to MEL-B (Fig. 1a). The efficacy of MEL-B was further confirmed using the BM1006 and JE2 MRSA strains via measurement of culture medium turbidity (OD_660_). Culture turbidity was significantly lower when either JE2 or BM1006 were grown in the presence of 32–512 ppm MEL-B (Fig. 1b) compared with that in untreated cultures (0 ppm). Moreover, consistent with a previous study that demonstrated the antimicrobial activity of MEL-B against most gram-positive (but not gram-negative) bacteria (Kitamoto et al. 1993), the growth of *E. coli* JM109, a representative gram-negative bacterium, was not significantly affected in the presence of MEL-B even at high concentrations (Fig. 2). These results support previous findings demonstrating the efficacy of MEL-B in inhibiting the growth of bovine-derived *S. aureus* (but not *E. coli*) and confirm the usefulness of measuring the CFU counts in our culture conditions to assess the roles of MEL-B.

### MEL-B is stable during in vitro culture

**Fig. 3:**
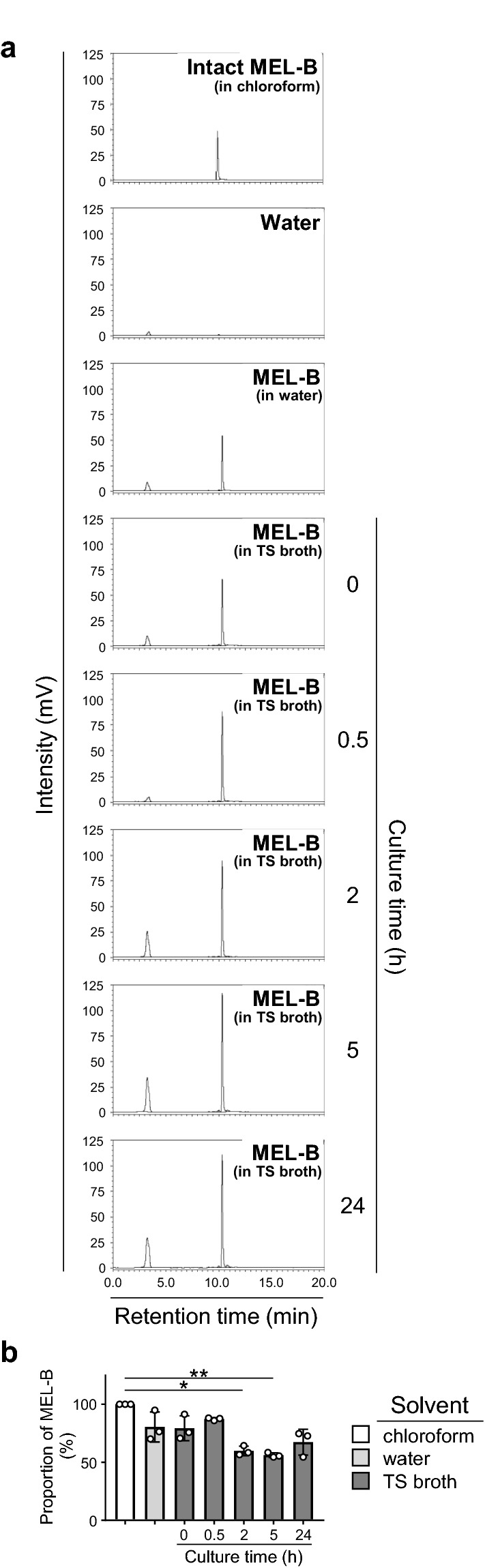
MEL-B is stable during culture in TS broth. **a** *S. aureus* BM1006 was cultured in the presence of MEL-B at 1000 ppm for 0.5, 2, 5, and 24 h. HPLC retention times of MEL-B in culture media were compared with those of fresh MEL-B undiluted or diluted with water or TS broth. In addition to the typical peak corresponding to MEL-B, another peak with a shorter retention time was detected when MEL-B was dissolved in water or TS broth, and the ratio of peak with a shorter retention time increased during culturing. **b** The proportion (%) of MEL-B in each condition was calculated by comparing the original peak corresponding to the intact MEL-B peak in chloroform. Statistical analyses were conducted using one-way ANOVA with the Kruskal–Wallis test. *p < 0.05, **p < 0.01

Although MEL-B possessed antimicrobial activity against *S. aureus*, the growth of *S. aureus* gradually continued during culture even in the presence of MEL-B at high concentrations. Therefore, we next addressed the condition of MEL-B during culture. Normal phase HPLC analysis showed that two peaks were present, one of which corresponded to intact MEL-B, whereas the other eluted at an earlier retention time corresponded to unknown molecule(s) excluded by HPLC separation because of low polarity (Fig. 3a). Importantly, the retention time of MEL-B did not change during culture with *S. aureus*, indicating that MEL-B remained intact in our culture conditions. The first peak with a shorter retention time was also slightly but clearly apparent even in water (Fig. 3a). Furthermore, the amount of this initial peak increased during culture with *S. aureus*, resulting in a decrease in the proportion of MEL-B (Fig. 3b). These results suggest that hydrophobic molecule(s) produced by *S. aureus* may influence the antimicrobial effect of MEL-B in vitro.

### MEL-B associates with the cell surface of both *S. aureus* and *E. coli*

**Fig. 4:**
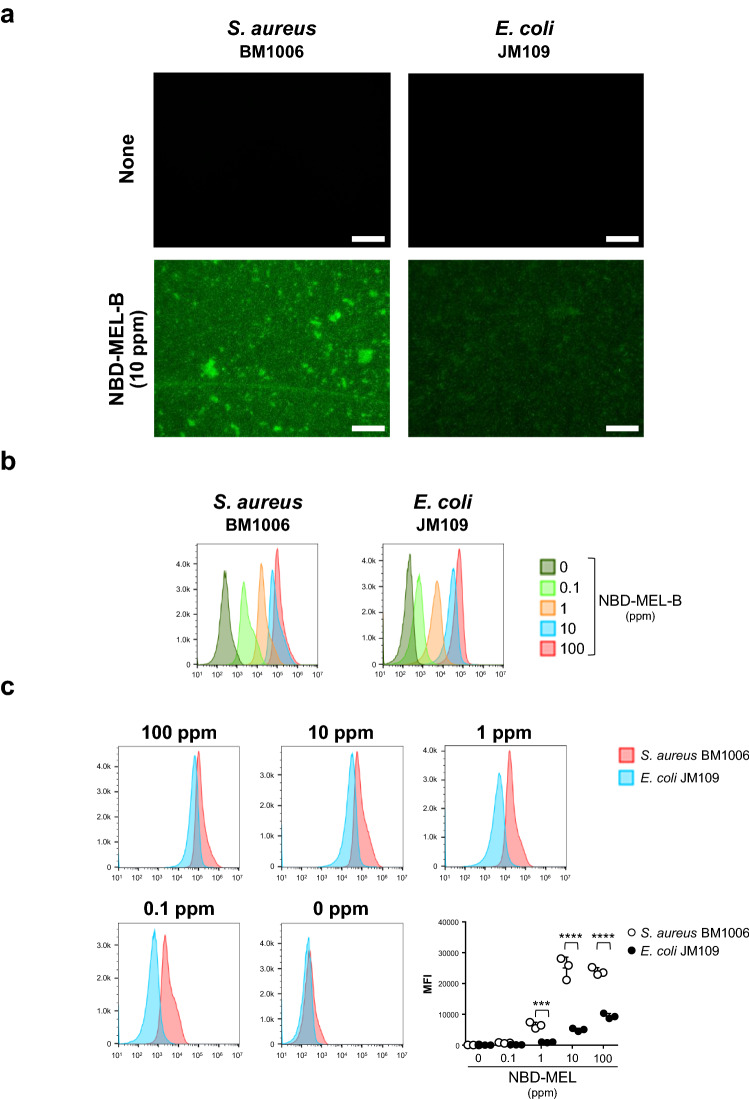
MEL-B binds to the surface of both *S. aureus* BM1006 and *E. coli* JM109 with different binding affinities. **a** Fluorescence signals were detected from *S. aureus* and *E. coli* when bacteria were incubated for 30 min in vitro with MEL-B that was pre-conjugated with a fluorescence molecule NBD (NBD-MEL-B). No fluorescence signals were seen from untreated *S. aureus* or *E. coli*. Representative images obtained using 10 ppm of NBD-MEL-B were shown. **b** Flow cytometric analysis at 0, 0.1, 1, 10, or 100 ppm of MEL-B showed that NBD-MEL-B associated with *S. aureus* and *E. coli* in a dose-dependent manner. **c** Mean fluorescence intensity (MFI) obtained from *S. aureus* was higher than that obtained from *E. coli* when bacteria were individually incubated with NBD-MEL-B above 1 ppm. Experiments were performed in triplicate. The representative data were shown in histograms and all MFIs obtained from three experiments were summarized in a dot graph. Statistical analyses were conducted using *t*-test. Scale bars = 50 μm. ***p < 0.001, ****p < 0.0001

To understand the molecular mechanism whereby MEL-B inhibits the growth of *S. aureus*, we labeled MEL-B with NBD and used this in a binding assay to confirm the association between MEL-B and *S. aureus*. The incubation of *S. aureus* with 10 ppm of NBD-labeled MEL-B (NBD-MEL-B) made the bacterial surface clearly fluorescent under a microscope (Fig. 4a). Interestingly, *E. coli* JM109 treated with the same concentration of NBD-MEL-B displayed weak (but apparent) fluorescence despite the lack of inhibitory effect of MEL-B on the growth of *E. coli* (Fig. 4a). No fluorescence was seen in either untreated *S. aureus* or *E. coli* (Fig. 4a). We confirmed this result using flow cytometry analysis with *S. aureus* and *E. coli*, both of which were treated with NBD-MEL-B at various concentrations (0, 0.1, 1, 10, and 100 ppm). The analysis indicated that NBD-MEL-B was associated with both *S. aureus* and *E. coli* in a dose-dependent manner (Fig. 4b). Furthermore, the fluorescence intensities observed in *S. aureus* were significantly higher than those in *E. coli* at NBD-MEL-B concentrations of 1 ppm or higher (Fig. 4c). These results suggest that the actions mediated by the binding of MEL-B to *S. aureus* (rather than simply the act of binding) may be necessary to inhibit bacterial growth.

### MEL-B does not damage the structure of *S. aureus*

**Fig. 5:**
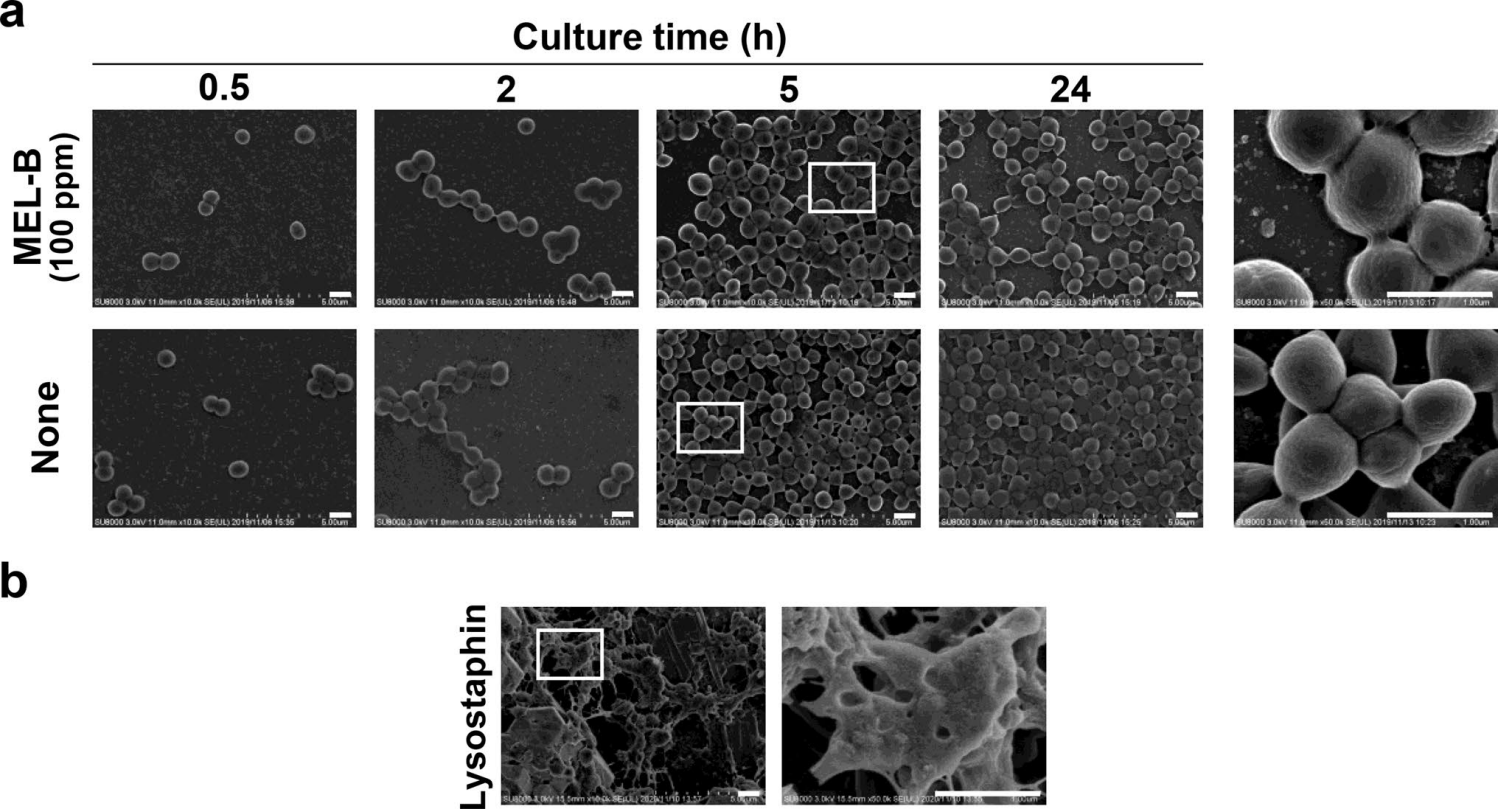
MEL-B does not disrupt the surface structure of *S. aureus* BM1006. **a** Scanning electron microscopic (SEM) analysis showed that the morphology of *S. aureus* did not change through in vitro culture in the presence of a relatively high concentration (100 ppm) of MEL-B. **b** *S. aureus* was treated with 100 U/mL of lysostaphin to confirm the disruption of the bacterial structure as a control in the SEM analysis. Scale bar = 1 μm

We then used SEM to chronologically examine the influence of MEL-B treatment on the structure of *S. aureus* during culture with MEL-B at relatively high concentration (100 ppm). Although MEL-B was anticipated to affect the surface structure of *S. aureus* by binding, no apparent differences could be seen in *S. aureus* during culture for 24 h with MEL-B when compared with *S. aureus* cultured without MEL-B (Fig. 5a). The typical shape of a grape-like cluster of *S. aureus* was observed regardless of MEL-B treatment (Fig. 5a). Moreover, these structures were completely different from those of damaged *S. aureus* treated with lysostaphin, which specifically disrupts the cell wall of *S. aureus* (Fig. 5b) (Watanakunakorn et al. 1971). These results suggest that the effect of MEL-B on the inhibition of the growth of *S. aureus* may not be due to the disruption of the overall bacterial structure.

### Fatty acids, components of MEL-B, are involved in inhibiting the growth of *S. aureus*

**Fig. 6:**
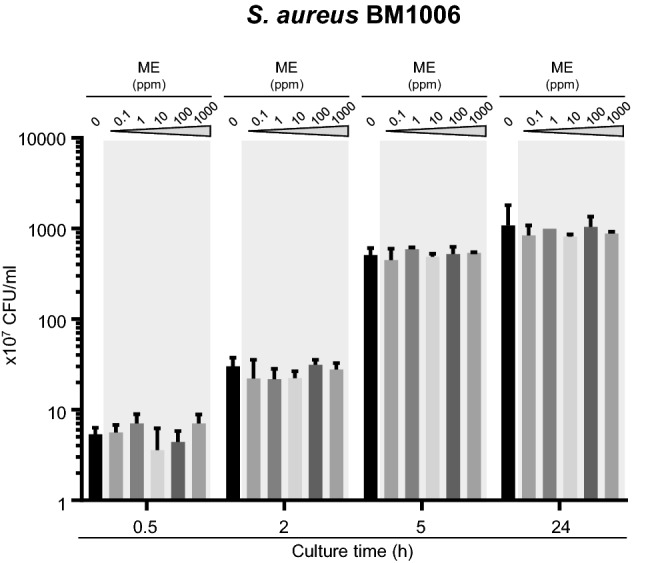
ME, a component of MEL-B, does not inhibit the growth of *S. aureus* BM1006 in vitro. *S. aureus* was cultured in the presence of ME at 0, 0.1, 1, 10, 100, or 1000 ppm for 0.5, 2, 5, and 24 h. The effect of ME on the growth of *S. aureus* was assessed by counting colony-forming units (CFUs). ME did not affect the growth of *S. aureus* even at high concentrations. Experiments were performed in triplicate, and the mean ± standard error of the mean obtained from representative data is shown. Statistical analyses were conducted using two-way ANOVA with Tukey’s multiple comparison test

Since MEL-B is composed of mannopyranose-erythritol (ME), which has a hydrophilic property, and two different fatty acids [i.e., caprylic acid (C8:0) and myristoleic acid (C14:1)], which have hydrophobic properties (Yamamoto et al. 2012), we then identified the critical components of MEL-B that affect the growth of *S. aureus*. Supplementation of ME, obtained as an intermediate product in MEL-B synthesis, to *S. aureus* culture at various concentrations (0, 0.1, 1, 10, 100, and 1000 ppm) did not have any inhibitory effects, even at high concentrations (Fig. 6). Unfortunately, the binding capability of ME to the cell surface of *S. aureus* could not be investigated because of the technical difficulty of synthesizing fluorescence-conjugated ME; however, this result suggested that the hydrophobic fatty acid components of MEL-B may directly or indirectly inhibit the growth of *S. aureus*.

### ME acts as a delivery vehicle for caprylic acid and myristoleic acid to *S. aureus*

**Fig. 7:**
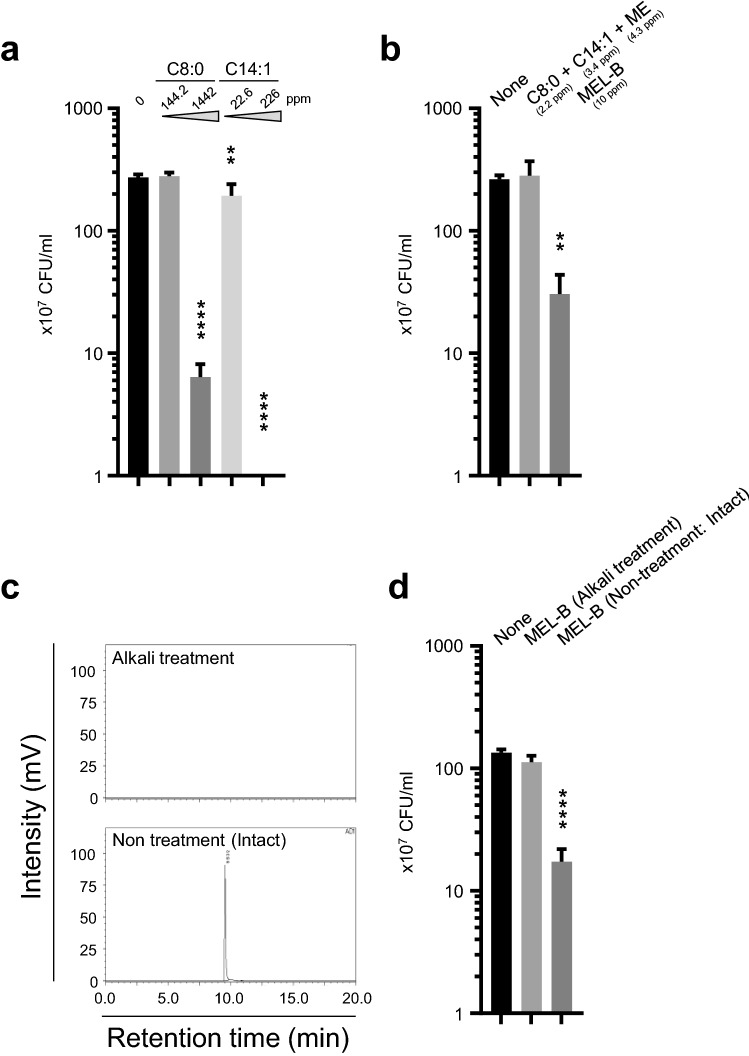
Modification of ME with two fatty acids is essential for effectively inhibiting the growth of *S. aureus* BM1006. **a** Caprylic acid (C8:0) and myristoleic acid (C14:1), both of which associate with ME, showed clear antimicrobial activity against *S. aureus* when cultured for 5 h at 1442 and 226 ppm, respectively. **b** A mixture of 4.3 ppm ME with 2.2 ppm caprylic and 3.4 ppm myristoleic acid, which were adjusted to the same concentration as 10 ppm MEL-B, did not inhibit the growth of *S. aureus*. **c** HPLC analysis showed that the peak corresponding to intact MEL-B disappeared when MEL-B was pre-treated in alkali solution. **d** Alkali-treated MEL-B, which had dissociated into MEL and two fatty acids, reduced the antibacterial activity against *S. aureus* when compared with non-treated intact MEL-B. Experiments were performed in triplicate, and the mean ± standard error of the mean obtained from representative data is shown. Statistical analyses were conducted using one-way ANOVA with the Kruskal–Wallis test. **p < 0.01, ****p < 0.0001

To elucidate the mechanism of action of MEL-B on the growth inhibition of *S. aureus*, three related experiments were conducted to clarify the functions of ME and fatty acids. Both caprylic and myristoleic acids are known to possess antimicrobial activity against *S. aureus* (Kabara, 1984). Therefore, we determined the minimum concentration of caprylic and myristoleic acids required to inhibit the growth of *S. aureus*. Since the efficacy of MEL-B in inhibiting the growth of *S. aureus* was clearly observed 5 h after culture (as shown in Fig. 1), *S. aureus* was cultured for 5 h in the presence of either caprylic acid (144.2 or 1442 ppm) or myristoleic acid (22.6 or 226 ppm). There was a significant decrease in CFU when 1442 ppm caprylic acid or 226 ppm myristoleic acid was added to the culture (Fig. 7a). We then compared the efficacy of MEL-B and a mixture of ME, caprylic acid, and myristoleic acid at the same concentration. The molecular weight of MEL-B composed of these components is 660.8 Da, and therefore, we used 10 ppm (the minimum dose of MEL-B required to inhibit the growth of *S. aureus*), which is equivalent to 0.015 mM. However, treatment of *S. aureus* with a mixture of 4.3 ppm ME, 2.2 ppm caprylic acid, and 3.4 ppm myristoleic acid, all of which were 0.015 mM, did not reduce *S. aureus* CFU (Fig. 7b). We then demonstrated the reduction of MEL-B efficacy by digestion of the ester bond that connects the ME with the two fatty acids. Specifically, complete digestion of 1000 ppm of MEL in alkali solution, which digests the ester bond, was confirmed via HPLC on the basis of the disappearance of the peak corresponding to MEL-B (Fig. 7c). Importantly, alkali-treated MEL-B did not inhibit the growth of *S. aureus* (Fig. 7d). These results indicated that the efficacy of MEL-B could be explained by the effective association of the antimicrobial caprylic and myristoleic acid components with the surface of *S. aureus* via the hydrophilic ME moiety.

## Discussion

Our study aimed to evaluate MEL-B as a possible disinfectant for use in the livestock industry to control the spread of *S. aureus* strains, including MRSA. We demonstrated that the growth of *S. aureus* BM1006, which belongs to the sequence type 352 that often causes mastitis in dairy cattle (Hata et al. 2010), is significantly inhibited when cultured in the presence of MEL-B in vitro. Notably, the pathogenicity of *S. aureus* BM1006 has been confirmed by in vivo infectious studies where mastitis was induced in dairy cattle when *S. aureus* BM1006 was inoculated into the udder (Nagasawa et al. 2018). A previous study has also shown that inflammatory chemokines (e.g., IL-8, CXCL6, and CCL2), which are involved in the recruitment of polymorphonuclear leukocytes into the udder, are abundantly secreted by mammary epithelial cells when stimulated with *S. aureus* BM1006 (Kiku et al. 2016). Given the virulence of *S. aureus* BM1006 as a causative bacteria of bovine mastitis, our results can provide insight into the development of a novel disinfectant to prevent the onset of mastitis in dairy cattle.

MEL-B had no effect on the growth of a representative gram-negative bacterium, *E. coli* JM109, in this study, which is consistent with previous results (Kitamoto et al. 1993). Our results also demonstrated that MEL-B bound to the cell surface of *E. coli* despite a lack of antimicrobial activity against the bacteria. Therefore, we hypothesized that further actions following the binding of MEL-B to the bacterial cell surface are required to inhibit the growth of gram-positive *S. aureus* (but not gram-negative *E. coli*). Recently, atomic force microscopy was used to demonstrate that the diameter of peptidoglycan pores can be up to 60 nm (Pasquina-Lemonche et al. 2020). Hence, MEL-B may diffuse freely into the cross-linked cell wall structure of gram-positive bacteria by penetrating the peptidoglycan interspaces and then interfering with the cell membrane, where functional molecules (e.g., transporters and receptors) are involved in nutrient uptake and signal transduction are expressed. In contrast, lipopolysaccharide found in the outer membrane of gram-negative bacteria may interfere with the free diffusion of MEL-B, leaving the cell membrane intact in these bacteria even after binding.

One of the significant advances obtained in this study was to elucidate how MEL-B inhibits the growth of *S. aureus*. Specifically, the ME moiety plays an important role in effectively exerting antimicrobial activity of the caprylic and myristoleic acids against *S. aureus*. Nevertheless, *S. aureus* still grew for 24 h when cultured in the presence of MEL-B at high concentration. A key structural feature of MEL-B is the ester bonding that connects ME and the two fatty acids, which is important for exhibiting antimicrobial activity. Our study showed that alkali-treated MEL-B, which was dissociated into ME and two fatty acids by digestion of ester bonds, did not inhibit the growth of *S. aureus*. Lipase produced by *S. aureus* during the culture was initially suspected as a factor that could reduce the antimicrobial activity of MEL-B. However, the peak corresponding to MEL-B was clearly observed in the medium even after culturing for 24 h, indicating that sufficient intact MEL-B remained in the culture throughout the experiment. Another possible hypothesis is that hydrophobic molecule(s) may affect the antimicrobial activity of MEL-B. HPLC analysis showed two major peaks corresponding to the MEL-B and unknown molecules(s) excluded by HPLC separation due to the low polarity (= high hydrophobicity). The peak with a shorter retention time was found even in water, and the amount increased during culture with *S. aureus*. These results suggest that the reduced antimicrobial activity of MEL-B against *S. aureus* during culture may be due to the effect of *S. aureus*-derived hydrophobic molecule(s) (but not impurities) in our culture conditions. Therefore, further studies are needed to identify appropriate solvent conditions that maintain or increase the efficacy of MEL-B for practical application as a disinfectant for dairy cattle.

A recent study demonstrated that the cell death of *S. aureus* was induced by treatment with MEL-A (Shu et al. 2020). By contrast, in our study using MEL-B, there was no sign to demonstrate apparent cell death, such as membrane disruption, although the growth of *S. aureus* was successfully inhibited by the treatment of MEL-B. This discrepancy may be due to the structural difference between MEL-A and MEL-B. MELs are divided into four types (i.e., MEL-A, MEL-B, MEL-C, and MEL-D), based on the number and position of the acetyl group (Saika et al. 2018), which depends on the microorganism responsible for synthesis (Coelho et al. 2020). One of the notable characteristics of MEL-A, which differentiates this from MEL-B, MEL-C, or MEL-D, is that MEL-A possesses an additional acetyl group, resulting in a higher hydrophobicity compared with that of the others (Imura et al. 2007; Saika et al. 2018). Since we did not compare the antimicrobial activity between MEL-A and MEL-B, further studies should be conducted to determine which form of MEL is most appropriate in controlling the spread of *S. aureus*. Nevertheless, it should be emphasized that MEL-B has been extensively used in the cosmetic industry, where it is used to suppress skin perspiration and increase water content (Yamamoto et al. 2012). Although verification is required to determine the use of MEL-B as a disinfectant for controlling mastitis caused by *S. aureus*, the safe use of MEL-B as a moisturizer for skin care should be positively appreciated for potential application in the dairy industry.

In conclusion, we elucidated the mechanism of action of MEL-B, composed of ME and antimicrobial fatty acids, in inhibiting the growth of bovine-derived mastitis causative *S. aureus*. ME acts as a delivery vehicle for antimicrobial fatty acids. Therefore, the binding between ME and fatty acids via an ester bond is essential for the effective transport of antimicrobial fatty acids that then associate with the surface of *S. aureus*. These results provide a novel insight into developing a disinfectant that could be used in farms to prevent dairy cattle from contracting mastitis.

## Data Availability

All data generated or analyzed during this study are included in this manuscript.
